# Integrated Microbiology and Metabolomics Analysis Reveal Responses of Soil Bacterial Communities and Metabolic Functions to N-Zn Co-Fertilization in the Rhizosphere of Tea Plants (*Camellia sinensis* L.)

**DOI:** 10.3390/plants14121811

**Published:** 2025-06-12

**Authors:** Min Lu, Yali Shi, Dandan Qi, Qiong Wang, Haowen Zhang, Ying Feng, Zhenli He, Chunwang Dong, Xiaoe Yang, Changbo Yuan

**Affiliations:** 1 State Key Laboratory of Nutrient Use and Management, Shandong Engineering Research Center of Tea Biology and Resource Utilization, Tea Research Institute, Shandong Academy of Agricultural Sciences, Jinan 250100, China; lumin@saas.ac.cn (M.L.); shiyali97@163.com (Y.S.); qidandan07@126.com (D.Q.); 2022120748@sdau.edu.cn (H.Z.); dongchunwang@163.com (C.D.); 2 School of Resources and Environment, Anqing Normal University, Anqing 246000, China; wq122506@zju.edu.cn; 3 Key Laboratory of Environmental Remediation and Ecosystem Health, Ministry of Education (MOE), College of Environmental and Resources Sciences, Zhejiang University, Hangzhou 310058, China; yfeng@zju.edu.cn; 4 Department of Soil, Water and Ecosystem Sciences, Indian River Research and Education Center, University of Florida-IFAS, Fort Pierce, FL 34945, USA; zhe@ufl.edu

**Keywords:** N-Zn co-fertilization, N availability, rhizosphere, bacterial community, metabolomics

## Abstract

The co-fertilization of nitrogen (N) and zinc (Zn) offers significant advantages in improving the growth and development of tea plants (*Camellia sinensis* L). However, the corresponding responses of rhizosphere microecology remain unclear. In this study, a pot experiment was performed to investigate the effects of N-Zn co-fertilization on rhizosphere soil’s N availability, the rhizobacterial community and the metabolism of tea plants. N-Zn co-fertilization significantly increased the soil total of N, NH_4_^+^-N and NO_3_^−^-N contents. 16S rRNA sequencing found that N-Zn co-fertilization recruited rhizobacteria associated with N cycling and Zn activation, including *Proteobacteria*, *Acidobacteriota* and *Gemmatimonadota*, resulting in complex rhizobacterial networks. Metabolomics analysis indicated obvious interferences in the metabolisms of lipids, amino acids and cofactors and vitamins after fertilization. PLS-PM analysis suggested that fertilization had both direct and indirect influences on the rhizobacterial community and differential metabolites. RDA models identified pH (R^2^ = 0.734, *p* < 0.01; R^2^ = 0.808, *p* < 0.01) and total N (R^2^ = 0.633, *p* < 0.05; R^2^ = 0.608, *p* < 0.01) as dominant factors influencing both the rhizobacterial community and differential metabolites. Finally, network analysis found significant associations between rhizobacteria related to N cycling and Zn mobilization and metabolic processes involved in N metabolism and responses to Zn stress. These findings underscored that appropriate N-Zn co-fertilization is crucial for the rhizosphere soil’s N availability and the microenvironment of tea plants.

## 1. Introduction

Tea is one of the most consumed manufactured drinks worldwide. The tea plant (*Camellia sinensis* L.) is a shrubby and perennial plant that thrives in acidic soil with an optimum pH of 4.0–5.51, and it is widely cultivated in tropical and subtropical regions [1]. With the rapid increase in tea consumption, China has emerged as the primary global producer, accounting for 62.1% of the total tea-planting area and 47.6% of tea production worldwide in 2020 [2,3]. To ensure the sustainable development of the tea industry and improve the yield and quality of tea, appropriate fertilization is an effective practice [4].

Nitrogen (N) is an essential nutrient element for plant survival and growth [5]. As a crucial constituent of biological molecules, such as proteins, nucleic acids, lipids and chlorophyll, N plays a pivotal role in stimulating the synthesis of C- and N-related compounds. Consequently, it significantly contributes to the yield and quality of tea plants [6]. In modern intensive agricultural systems, N fertilization is widely employed to enhance plant productivity and quality [7]. However, while promoting plant growth positively, an excessive N supply can also have adverse effects on soil ecosystems. These include soil acidification, greenhouse gas emissions and alterations in microorganism species diversity. Such negative impacts are attributed to the side effects of N transformation processes within soils [8]. Also, an excessive N supply has been found to promote the synthesis of arginine rather than theanine, resulting in a bitter taste and a decrease in tea quality [9]. Therefore, it is imperative to explore optimal methods for N fertilization that ensure sustainable development within intensive agricultural ecosystems [10].

Zinc (Zn) is an indispensable trace element in plant growth and development [11]. It serves as a constituent or activator for numerous enzymes, playing a pivotal role in substance hydrolysis, redox processes and protein synthesis within tea plants. For instance, it regulates sugar conversion in tea plants and facilitates the formation of quality components such as amino acids, catechins and aroma substances [12]. Recent studies have discovered the synergistic effect of Zn on soil N transformation processes in crop systems due to its cofactor role in certain N metabolism-related enzymes [13]. Xu et al. [14] manifested the functions of CsZIP4 in N utilization and Zn transport in tea plants. However, to date, there has been no report on the soil microecology mechanisms of the synergistic effect of N-Zn co-fertilization.

Soil microorganisms play pivotal roles in maintaining soil function and they exert significant influence on soil productivity and plant yield [15]. Microorganisms modulate soil nutrient cycling by stabilizing and decomposing organic matter, thereby impacting soil enzyme activities [16]. Previous studies have demonstrated that fertilization could alter the microbiological equilibrium, which is crucial for plant growth and soil health, through modifications of physicochemical properties, enzymatic profiles, microbial biomass and community composition. For example, long-term N fertilization experiments conducted in tea plantations revealed that increasing N rates led to significant changes in soil community characteristics including reduced diversity, weakened functional capacity and decreased stability [17]. Liu et al. [18] reported that optimal Zn application stimulated soil enzyme activities and induced shifts in the relative abundance of several bacterial taxa such as *Rhodospirillales*, *Gaiellales*, *Frankiales* and *Latescibacteria* after a nine-year field experiment in a wheat (*Triticum aestivum* L.)–maize (*Zea mays* L.) system. Conversely, results from N-Zn co-fertilization treatment illustrated that Zn application facilitated N transformation with improved availability by modifying microbial communities as well as altering soil enzyme activities and functional gene expression levels, ultimately promoting N uptake efficiency and biomass production in rice (*Oryza sativa* L.) [19].

To better elucidate the underlying mechanisms by which fertilizer availability influences the soil microenvironment more comprehensively, the alteration of soil metabolic function resulting from microbial community change has been investigated [20,21]. Additionally, the application of metabolomics technology enables the identification and characterization of alterations in soil metabolites and metabolic pathways after soil fertilization [22]. Cheng et al. [23] combined microbiology with non-target metabolomics using high-throughput sequencing and UPLC-MS/MS platforms to explore how phosphorus fertilizer levels and fertilization patterns affect wheat soil microbial communities and metabolic functions. However, there is still a lack of research on the effects of N-Zn co-fertilization on soil microbial metabolites, metabolic pathways and functions. Therefore, integrating the analysis of soil microbial communities with soil metabonomics will facilitate a more profound comprehension of the response and underlying mechanisms of the synergistic effect of N and Zn fertilization in soil [24].

In this study, the combined study of the physiochemical response, bacterial communities and metabolomics in the rhizosphere soil of tea plants in response to different combinations of N and Zn fertilization was carried out through a pot experiment. The objectives of this study were to (i) characterize the responses of soil N availability, microbial biomass, and enzyme activities in the rhizosphere of tea plants; (ii) elucidate the differential changes in soil bacterial communities and soil metabolic functions in the rhizosphere of tea plants and (iii) preliminarily explore the relationships among the soil physiochemical response, soil bacterial communities and soil metabolism.

## 2. Results

### 2.1. Soil Properties, Microbial Biomass and Enzyme Activities in the Rhizosphere of Tea Plants

**Figure 1:**
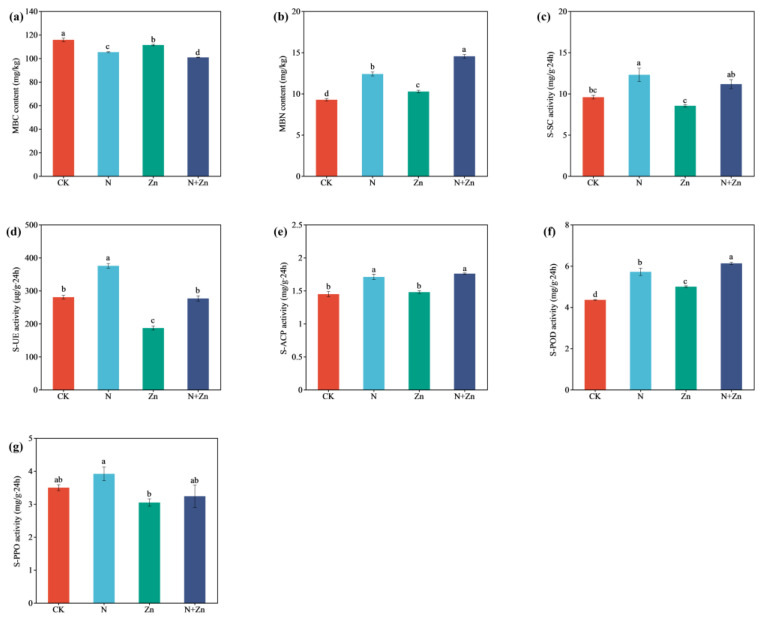
Rhizosphere soil microbial biomass carbon (MBC) contents (**a**), microbial biomass nitrogen (MBN) contents (**b**), soil sucrase (S-SC) activities (**c**), soil urease (S-UE) activities (**d**), soil acid phosphatase (S-ACP) activities (**e**), soil peroxidase (S-POD) activities (**f**) and soil polyphenol oxidase (S-PPO) activities (**g**) of tea plants (*Camellia sinensis* L.) in response to different combinations of N and Zn fertilization. Different letters above bars indicate significant differences among all treatments at *p* < 0.05.

Compared to CK, both Zn and N+Zn treatments significantly altered the rhizosphere soil’s total Zn (TZn) content (increased by 1.14- and 1.16-fold, respectively) as well as the DTPA-Zn (AZn) content (increased by 3.67- and 1.74-fold, respectively) (Table 1). Similarly, fertilization had a significant impact on the rhizosphere soil’s physiochemical properties. The N and N+Zn treatments notably enhanced the rhizosphere soil’s total N content (TN) (by 58.1% and 52.8%, respectively), NO_3_^−^-N content (by 140% and 150%, respectively), NH_4_^+^-N content (by 234% and 229%, respectively) and microbial biomass nitrogen (MBN) content (by 33.7% and 56.9%, respectively), but decreased the pH value (by 10.8% and 8.76%, respectively), organic matter content (OM) (by 28.1% and 37.3%, respectively) and microbial biomass carbon (MBC) content (by 9.01% and 12.9%, respectively) of the rhizosphere soil, as compared to CK (Table 1 and Figure 1a,b). Apart from soil peroxidase (S-POD) and soil polyphenol oxidase (S-PPO), the activities of other enzymes in the rhizosphere soil were also affected by exogenous fertilizer application (Figure 1). In comparison with CK, N treatment significantly increased the activities of soil urease (S-UE), soil acid phosphatase (S-ACP) and soil sucrase (S-SC) in the rhizosphere, with values of 33.8%, 18.4% and 28.4%, respectively. The activity of S-UE was reduced by 33.3% to CK after Zn fertilization but reduced by 26.3% to N treatment after N+Zn co-fertilization (Figure 1c–e).

### 2.2. Soil Rhizobacterial Communities of Tea Plants

**Figure 2:**
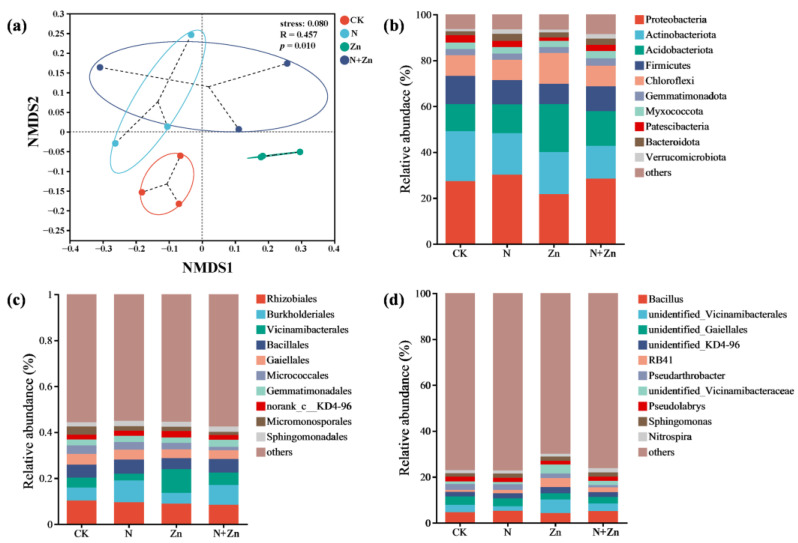
Soil rhizobacterial community analysis in response to different combinations of N and Zn fertilization. NMDS analysis (**a**) of the rhizobacterial community for visualization of Bray-Curtis distances in response to different combinations of N and Zn fertilization. Relative abundance of top 10 rhizobacterial phyla (**b**), orders (**c**) and genera (**d**) in response to different combinations of N and Zn fertilization.

To assess the diversity and richness of the rhizobacterial community (Figure S1), α-diversity indexes were calculated based on the OTU level. Overall, although fertilization had an impact on soil rhizobacterial community diversity compared to CK, these differences were not statistically significant (*p* > 0.05). Notably, N+Zn treatment showed higher rhizobacterial community diversity than N or Zn treatment. Variations in the rhizobacterial community across four treatments were evaluated by NMDS analysis and PERMANOVA analysis (R^2^ = 0.399, *p* < 0.05). NMDS analysis based on Bray-Curtis (stress = 0.080, R = 0.457, *p* < 0.05) revealed that there was obvious dissimilarity across the four treatments (Figure 2a). With regards to the rhizobacterial community compositions, the bacterial sequences were distributed in 44 phyla, 596 families, 148 classes and 1184 genera. The phyla *Proteobacteria*, *Actinobacteria*, *Acidobacteria*, *Firmicutes* and *Chloroflexi* accounted for up to 53% of the bacterial communities (Figure 2b). Compared with CK, the relative abundances (RAs) of *Acidobacteria* and *Chloroflexi* were significantly increased under Zn treatment, which also significantly decreased the RAs of *Proteobacteria*, *Firmicutes* and *Patescibacteria* (Kruskal-Wallis test, *p* < 0.05; Figure S2). The top 10 orders of rhizobacteria accounted for more than 50% of all sequences, including *Rhizobiales*, *Burkholderiales*, *Vicinamibacterales*, *Bacillales*, *Gaiellales*, *Micrococcales*, *Gemmatimonadales*, *norank_c__KD4-96*, *Micromonosporales* and *Sphingomonadales* (Figure 2c). At the genus level, the top 10 genera of rhizobacteria were *Bacillus*, *norank_f__norank_o__Vicinamibacterales*, *norank_f__norank_o__Gaiellales norank_f__norank_o__norank_c__KD4-96*, *RB41*, *Pseudarthrobacter*, *norank_f__Vicinamibacteraceae*, *Pseudolabrys*, *Sphingomonas* and *Nitrospira*, which represented about 25% of all sequences (Figure 2d). The RA of RB41 under Zn treatment were increased while the RA of *Pseudolabrys* were decreased in comparison with CK (Kruskal-Wallis H test, *p* < 0.05; Figure S4).

**Figure 3:**
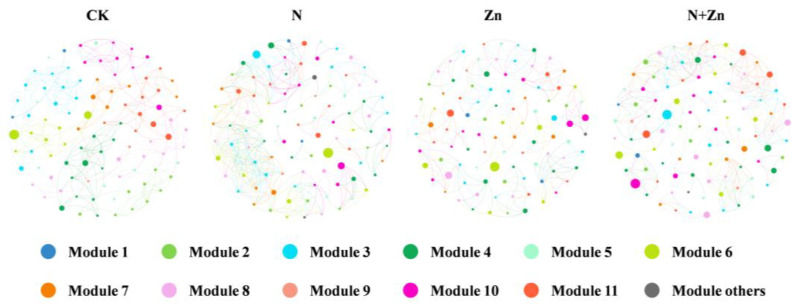
Effect of different combinations of N and Zn fertilization on the co-occurrence patterns of soil rhizobacterial community. Networks were constructed at the operational taxonomic unit (OTU) level. The size of nodes (OTUs) is scaled to the degree of nodes, and the nodes are colored according to modules. Edges indicate correlations among nodes (|R| > 0.6, *p* < 0.05).

Considering the crucial role of biological interactions in shaping the rhizobacterial community structure, we constructed soil bacterial co-occurrence networks across all treatments to better illustrate the interplay among soil rhizobacterial communities (Figure 3). The results revealed altered co-occurrence patterns and changes in the organization of rhizobacterial communities due to fertilization. Specifically, compared to CK, both N and N+Zn treatments exhibited significantly higher complexity and connectivity in their soil networks, as evidenced by the increased average degree, number of network nodes, number of network edges and average clustering coefficient (Table 2). Conversely, Zn treatment displayed lower complexity and connectivity along with the lowest proportion of negative correlations compared to CK.

### 2.3. Soil Metabolic Profile in the Rhizosphere of Tea Plants

**Figure 4:**
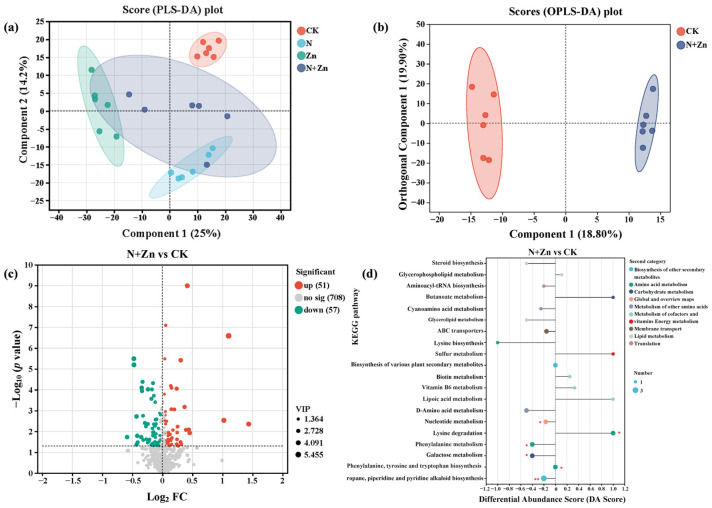
Rhizosphere soil metabolomics analysis of tea plants in response to different combinations of N and Zn fertilization. PLS-DA score plots derived from metabolites in response to different combinations of N and Zn fertilization (**a**). OPLS-DA score plots derived from metabolites between CK and N+Zn treatment (**b**). The expression volcano map of differential metabolites up and down regulation between CK and N+Zn treatment (**c**) (Green dots represent down-regulated metabolites, red dots represent up-regulated metabolites, and gray dots represent no-differential metabolites.). Differential abundance score of KEGG metabolic pathways between CK and N+Zn treatment (**d**) (** indicates significance at *p* < 0.01. * indicates significance at *p* < 0.05).

To establish the comprehensive metabolic profile in the rhizosphere soil of tea plants after different combinations of N and Zn fertilization, we conducted non-targeted metabolomics using UHPLC-MS/MS. Overall, a total of 6604 peaks and 816 metabolites responsive to fertilization were detected across all of the rhizosphere soil samples. PLS-DA results demonstrated distinct separations after fertilization for all samples, explaining 25% (component **1**) and 14.2% (component **2**) of the total variance, respectively (Figure 4a). Further, based on the criteria of the VIP scores (VIP > 1) from the OPLS-DA model as well as the student *t*-test (*p* < 0.05), many differential metabolites (DEMs) were identified (Figure 4b, Figures S5a and S6a). A total of 227, 185 and 108 DEMs were identified in the rhizosphere soil samples under N, Zn and N+Zn treatments as compared to CK, respectively (Figure 4c, Figures S5b, S6b and S7).

**Figure 5:**
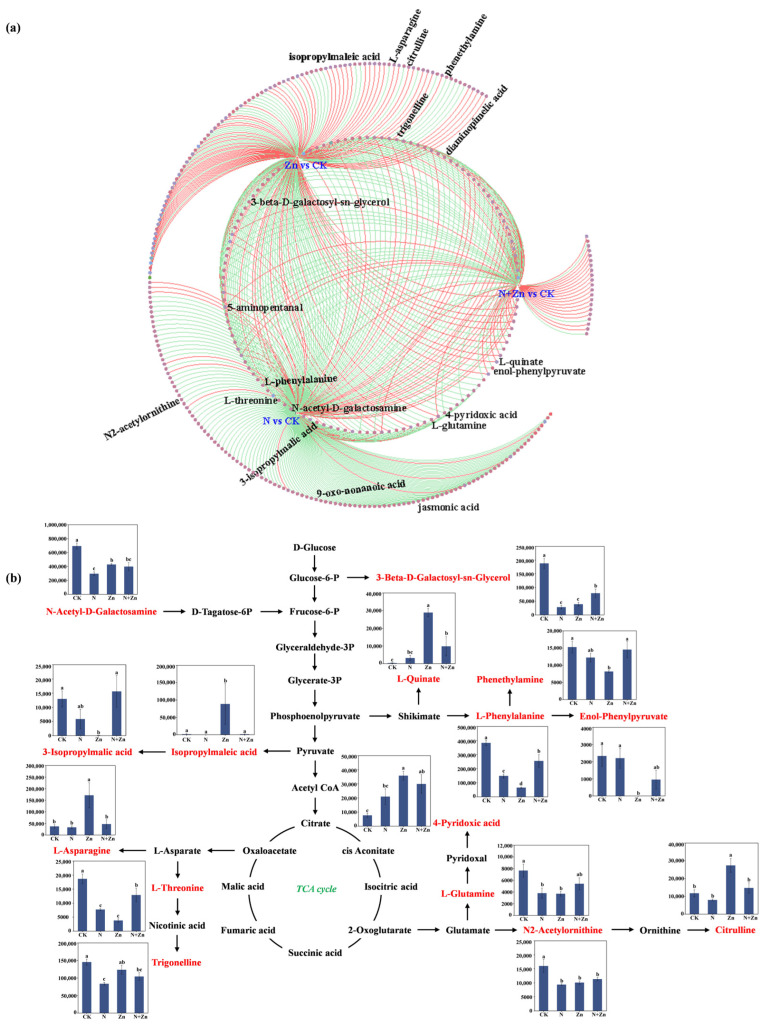
Collinearity analysis of different marker metabolites in rhizosphere soil in response to different combinations of N and Zn fertilization. The circles represent metabolites, and the shade of color indicates the size of the difference multiple; the red and green lines represent the increase or decrease of metabolites in the corresponding group (**a**). Metabolic pathway map of differential marker metabolites in rhizosphere soil in response to different combinations of N and Zn fertilization. Histogram shows the relative abundance of 15 DEMs across the different treatments (**b**). Different letters above bars indicate significant differences among all treatments at *p* < 0.05.

Enrichment analysis of KEGG pathways revealed that each treatment showed enrichment in 13, 14 and 6 differential metabolic pathways, respectively (Figure 4d, Figures S5c and S6c). Interestingly, 10 common potential pathways were identified in two or three treatments, including the Nucleotide metabolism, Vitamin B6 metabolism, Arginine biosynthesis, Phenylalanine, tyrosine and tryptophan biosynthesis, Valine, leucine and isoleucine biosynthesis, Phenylalanine metabolism, Pyrimidine metabolism, Tropane, piperidine and pyridine alkaloid biosynthesis, Aminoacyl-tRNA biosynthesis and Purine metabolism. These findings indicated a significant impact of fertilization on the synthesis of metabolites and their corresponding metabolic pathways. In addition, through the collinearity analysis, changes in the DEMs under different fertilization treatments were clarified (Figure 5a). To further investigate the impact of fertilization on the rhizosphere soil metabolism of tea plants, we constructed a metabolic pathway consisting of 15 DEMs by referencing the KEGG pathway database (Figure 5b). The levels of these DEMs significantly varied across all treatments. Specially, L-glutamine, L-threonine, N2-acetylornithine, L-phenylalanine, enol-phenylpyruvate, phenethylamine, trigonelline, 3-beta-D-galactosyl-sn-glycerol and N-acetyl-D-galactosamine were significantly decreased after fertilization, with all except L-glutamine and N2-acetylornithine significantly varying among the fertilizer treatments. Among them, L-glutamine, L-threonine, L-phenylalanine and phenethylamine had the least content after Zn treatment, with 2.07-fold, 4.91-fold, 5.89-fold and 1.87-fold more down-regulations than CK, respectively. All of the fertilizer treatments significantly increased the levels of L-quinate, which reached the maximal value under Zn treatment (107.6 times the control value). Additionally, the levels of 3-beta-D-galactosyl-sn-glycerol and N-acetyl-D-galactosamine, involved in Galactose metabolism, were altered with 6.61-fold and 2.34-fold more down-regulations than CK, respectively (Figure 5b).

### 2.4. Correlations Between Soil Properties, Soil Rhizobacterial Community and Rhizosphere Soil Metabolism

**Figure 6:**
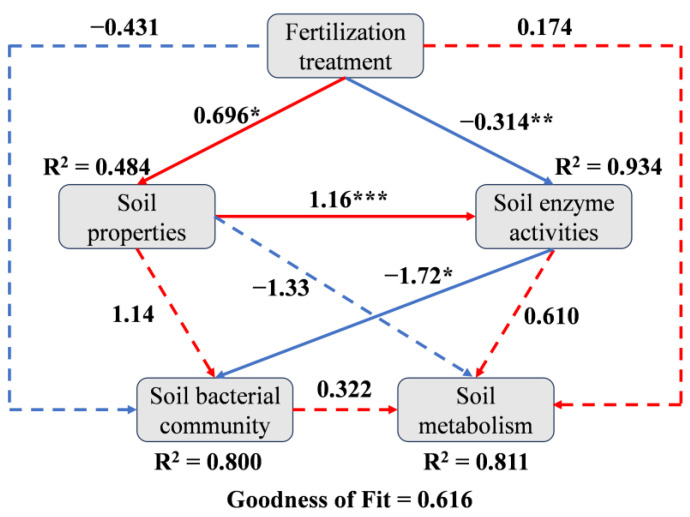
PLS-PM analysis showed the relationship between fertilization treatment, soil properties, soil enzyme activities, soil bacterial community and soil metabolism (goodness of fit = 0.616). The red and blue lines represent positive and negative effects, respectively. Solid and dashed lines indicate significant and non-significant effects, respectively. The values of R^2^ round the boxes represent the explanatory degree of the variables. The number on the line represents the total effect value (statistical significance is denoted by *, ** and *** for *p*-values less than 0.05, 0.01 and 0.001, respectively.).

**Figure 7:**
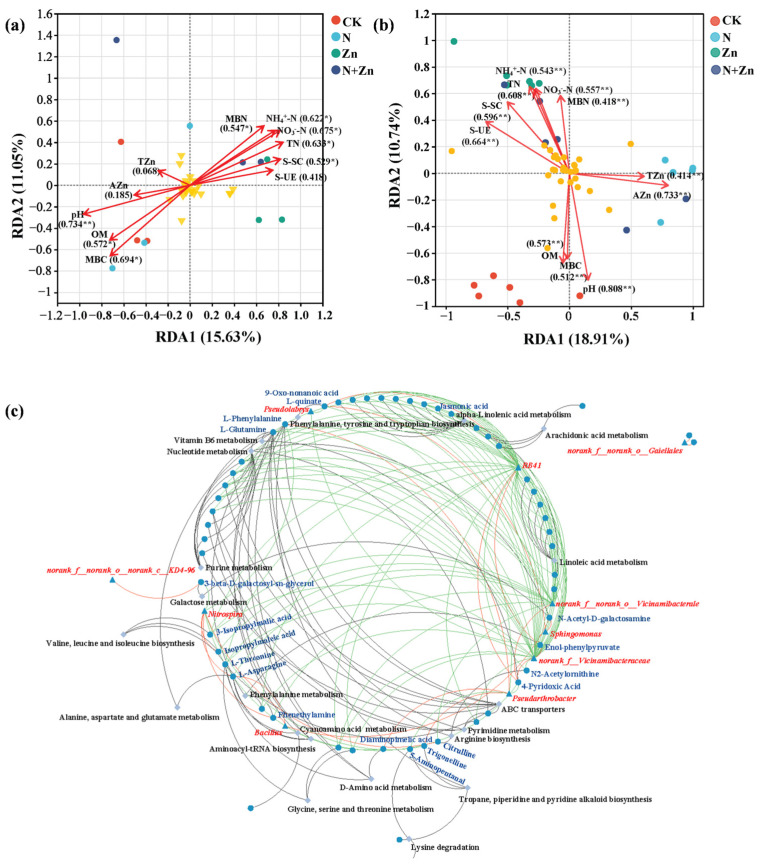
RDA analysis (**a**,**b**) of rhizosphere soil physicochemical properties and rhizobacterial community, DEMs (** indicates significance at *p* < 0.01. * indicates significance at *p* < 0.05). Interaction network diagram of the spearman correlation between rhizobacteria, DEMs and metabolic pathways in rhizosphere soil of tea plants (**c**) (the red and green lines represent positive and negative correlations between the rhizobacteria and DEMs. Triangles represent species at the genus level, circles represent metabolites and diamonds represent metabolic pathway).

A partial least squares path model (PLS-PM) analysis with a higher value of goodness of fit (0.616) was conducted to investigate the potential relationships between fertilization treatment, soil properties, soil enzyme activities, the soil rhizobacterial community and rhizosphere soil DEMs (Figure 6). The results indicated that fertilization treatment had a direct negative effect on the soil rhizobacterial community but a direct positive effect on rhizosphere soil DEMs with non-significant difference. Fertilization treatment had direct influences on soil properties and soil enzyme activities, which in turn directly affected the soil rhizobacterial community. The soil rhizobacterial community, in turn, had a direct positive effect on rhizosphere soil DEMs. Fertilization treatment affected rhizosphere soil DEMs indirectly by altering soil properties, soil enzyme activities and the soil rhizobacterial community. In summary, the PLS-PM results demonstrate that fertilization treatment altered rhizosphere soil DEMs by influencing soil properties and soil enzyme activities, and ultimately affecting the soil rhizobacterial community. Specifically, redundancy analysis (RDA) models were performed to assess the relationships between soil properties and soil enzyme activities with the soil rhizobacterial community, and rhizosphere soil DEMs. The RDA model depicting the relationship between soil properties and soil enzyme activities with the rhizobacterial community showed that the contribution rates for the eigenvalues on the RDA1 and RDA2 axes reached 15.63% and 11.05%, respectively (Figure 7a), while the two values of the RDA model depicting the relationship between soil properties and soil enzyme activities with rhizosphere soil DEMs reached 18.91% and 10.74%, respectively (Figure 7b). Soil pH, TN, NO_3_^−^-N, NH_4_^+^-N and MBC were the top five environmental factors influencing the rhizobacterial community, while, pH, AZn, S-UE, TN and S-SC were mainly involved in the regulation of the rhizosphere soil metabolism.

In addition, Spearman correlation methods were employed to screen the members responsible for the observed manipulations on the rhizobacteria. Significant correlations were observed linking the rhizobacterial genera, DEMs and the related metabolic pathways (Figure 7c). There were some rhizobacterial genera, such as the genera *Pseudolabrys*, *RB41*, *norank_f__Vicinamibacteraceae* and *norank_f__norank_o__Vicinamibacterales*, that are significantly correlated with multiple DEMs. Also, it was noted that some DEMs showed a significantly strong correlation with multiple rhizobacterial genera, including endol-phenylpyruvate, citrulline, L-quinate, phenethylamine, L-asparagine, isopropylmaleic acid, 4-pyridoxic acid, L-threonine, L-phenylalanine and jasmonic acid. In addition, after further association with metabolic pathways, rhizobacteria that may make important contributions to related metabolic processes were unearthed. Rhizobacterial genera that were significantly correlated with processes such as amino acid metabolism and the metabolism of cofactors and vitamins like *norank_f__norank_o__Vicinamibacterales*, *norank_f__norank_o__Gaiellales*, *RB41*, *norank_f__Vicinamibacteraceae*, *Pseudolabrys* and *Nitrospira* were also obtained.

## 3. Discussion

### 3.1. Alterations of Rhizosphere Soil N Availability of Tea Plants in Response to Different Combinations of N and Zn Fertilization

Previous studies have demonstrated that chemical fertilizers often lead to alterations in soil physicochemical characteristics [25]. This observation is consistent with our findings, which indicates significant changes in rhizosphere soil properties due to different combinations of N and Zn fertilizers, especially in soil N availability (Table 1). Glass and Orphan [13] found that the presence of Zn in soils exerts a synergistic impact on N turnover as it serves as a cofactor for numerous enzymes involved in N metabolism. In the present study, Zn fertilization did not influence rhizosphere soil N contents. However, both N fertilization and N-Zn co-fertilization significantly affected total N content and N availability of rhizosphere soil (Table 1). Although tea plants prefer NH_4_^+^-N uptake to support their N metabolism needs [9], both NH_4_^+^-N and NO_3_^−^-N contents in rhizosphere soil increased after N addition. And this phenomenon could be finally put down to the higher N addition under N and N+Zn treatments, which was recommended as 119–285 kg N/ha for tea plantation in China [17]. When excess urea was added to the soil, more free NH_4_^+^ were accumulated in soil solution under the catalysis of S-UE. After the urea hydrolysis, part of the free NH_4_^+^ were converted into NH_3_ and released into the air, while most of the free NH_4_^+^ were oxidized to NO_3_^−^ under the action of nitrification, resulting in the accumulation of NO_3_^−^-N in soil solution [26]. Consistent with the above deduction, rhizosphere soil MBN and the activities of S-UE were improved under N and N+Zn treatments (Figure 1). Also, positive correlations were observed between soil MBN, S-UE activities and soil total N content, NH_4_^+^-N content, NO_3_^−^-N content (Table S1).

### 3.2. Modifications of Soil Rhizobacterial Diversity and Communities of Tea Plants in Response to Different Combinations of N and Zn Fertilization

Soil microorganisms, particularly bacteria, exhibit remarkable abundance and diversity, playing pivotal roles in agricultural ecosystems through their active involvement in the nutrient cycling process, maintenance of soil structure, and facilitation of plant growth [27]. Previous studies have reported that the application of chemical fertilizers can alter the diversity and community structure of rhizosphere microorganisms. In this study, different combinations of N and Zn fertilizers influenced the diversity and richness of the soil rhizobacterial community without significant differences observed (Figure S1), which is consistent with previous findings [19]. However, variations were observed in both the structure and composition of the soil rhizobacterial community under different combinations of N and Zn fertilization (Figure 2). *Proteobacteria* and *Bacteroidota*, showing plant growth-promoting activity and N cycling, were found to be abundant in soils treated with N fertilizer (Figure 2b), evidenced by the positive associations of *Proteobacteria* and *Bacteroidota* with soil TN and NH_4_^+^-N and NO_3_^−^-N contents (Table S2). For example, previous studies have demonstrated that *Burkholderiales* (class *Gammaproteobacteria*) and several species of *Sphingomonas* could participate in N cycling and stimulate plant growth by the production of the hormone indole-3-acetic acid [28,29]. Rhizosphere of the tea plants under Zn treatment was mainly colonized by some taxa involved in organic matter degradation and metal mobilization, such as *Acidobacteriota* and *Chloroflexi* (Figure 2b). For example, *Vicinamibacterales* (phylum *Acidobacteria*), specially for *norank_f__norank_o__Vicinamibacterales*, and *norank_f__Vicinamibacteraceae*, which was thought to be tolerant to Zn stress and able to degrade organic matters and then produce organic acids [19,30], showed positive correlations with soil TZn and AZn contents (Tables S3 and S4) and might participate in the response to Zn stress and the degradation of organic matter from root exudates and rhizodeposits of the tea plants under Zn treatment. Interestingly, N+Zn treatment resulted in increased recruitment of diverse rhizobacterial taxa, including *Proteobacteria*, *Acidobacteriota* and *Gemmatimonadota*, participating both N cycling and Zn activation. Symbiotic networks play a pivotal role in maintaining the stability of microbial communities under external disturbances [31]. Previous studies have demonstrated that alterations in environmental conditions, can induce changes in the complexity of soil microbial networks [17,23,25]. The present study also revealed that different combinations of N and Zn fertilization influenced the complexity of the rhizobacterial network (Figure 3 and Table 2). N addition positively affected soil bacterial communities, with the increase of the complexity of rhizobacterial co-occurrence networks evaluated by higher values of number of edges and average degree. This result is contrary to that of Ma et al. [17] who found that excess N addition simplified soil bacterial community of tea plants. This could be probably due to the various dimensional scale of research. Additionally, compared to CK, both N and N+Zn treatments exhibited a higher proportion of positive interactions and average clustering coefficient, indicating enhanced cooperation among microbes and subsequent increased resistance to external disturbances [32,33].

### 3.3. Responses of Rhizosphere Soil DEMs and Potential Metabolic Pathways of Tea Plants in Response to Different Combinations of N and Zn Fertilization

Soil metabolites primarily originate from plant roots and microorganisms, with the composition of root exudates varying based on plant species, genotype, and environmental stress factors [34,35]. Changes in soil metabolite composition and content can provide insights into the direct or historical response of soil microorganisms to soil nutrients [36]. The results indicated that different combinations of N and Zn fertilization influenced rhizosphere soil metabolites of tea plants. These changes subsequently interfered with metabolic pathways related to N assimilation, carbon metabolism, and lipid metabolism. Specifically, the levels of L-threonine and L-phenylalanine were reduced after fertilization as compared to CK, while N+Zn treatment significantly increased their levels when compared to N or Zn treatments. The observed increase in these two amino acids suggested a disturbance in N metabolism due to co-fertilization with N and Zn fertilizer or substantial protein degradation. Additionally, L-glutamine and L-asparagine, two important amides, were also found altered following different combinations of N and Zn fertilization. L-glutamine and L-asparagine are the primary N-rich amino acids in leaves and play a crucial role in assimilating inorganic N [37]. The alterations observed in these two amino acids indicated the potential disturbances to the processes of N cycling. Furthermore, L-quinate, L-phenylalanine, phenethylamine, and enol-phenylpyruvate serve as intermediates within the shikimic acid pathway, which is pivotal for synthesizing aromatic amino acids essential for protein synthesis and acts as a critical source of precursors for diverse secondary metabolites [38]. Interestingly, the application of Zn fertilizer resulted in an increase in L-quinate levels but a decrease in phenylalanine, phenethylamine, and enol-phenylpyruvate levels. These findings indicate that elevated Zn concentrations may promote secondary metabolite synthesis, consequently leading to reduced precursor levels. Additionally, Zn fertilization also led to an elevation of the level of jasmonic acid, a compound known to play a significant role in plant growth and metabolism under heavy metal stress conditions [39]. Collectively, all the above changes in soil DEMs and the corresponding metabolic pathways in response to different combinations of N and Zn fertilization remained consistent with the alterations in soil rhizobacterial communities. And this evidence supports the notion that different combinations of N and Zn fertilization could effectively enhance plant-rhizobacterial metabolism.

### 3.4. Correlations Between Soil Properties, Bacterial Communities and DEMs in the Rhizosphere of Tea Plants in Response to Different Combinations of N and Zn Fertilization

Soil ecological functions are closely related to factors such as soil microbial community and soil metabolism, which could be shaped by soil properties and soil enzyme activities [35,40]. Aligning with previous research, in the present study, PLS-PM analysis suggests that fertilization treatment has direct influences on soil properties and soil enzyme activities, which in turn directly affects the soil rhizobacterial community, and ultimately affects rhizobacterial soil DEMs. Also, the RDA models demonstrate that the soil pH (R^2^ = 0.734, *p* < 0.01; R^2^ = 0.808, *p* < 0.01) and TN (R^2^ = 0.633, *p* < 0.05; R^2^ = 0.608, *p* < 0.01) are two dominant environmental factors influencing not only rhizobacterial communities but also soil metabolism in the rhizosphere of tea plants, corroborating previously reported effects [17]. It is generally accepted that soil pH is a critical factor that affects the soil microbial community [41]. Soil pH could change the acidity and the bioavailability of metal ions in the soil and then lead to variations in abundances of the acidity- and Zn-sensitive bacteria, thus affecting the microbial communities and soil metabolism [42]. As the building block of proteins and nucleic acids, soil N is essential for the growth of tea plants and microorganisms. N supply leads to alterations of soil N contents, and then changes the soil metabolism processes by influencing the stability and metabolic rate of soil ecosystems. Therefore, the enrichment of bacterial taxa related to N cycling and the alterations of soil N metabolism after N addition, as well as the colonization of bacterial taxa responsible for Zn activation and the up-regulation of soil DEMs in response to Zn stress under Zn addition, might be explained by the dominance of soil pH and TN across all the environmental factors. Noteworthy, soil microorganisms play a pivotal role in soil metabolic activities, thereby directly reflecting the response of soil microorganisms’ biological reactions to the soil environment. Moreover, alterations in microbial species and abundance partially regulate changes in soil metabolites, subsequently influencing the circulation and metabolism of soil nutrients [23]. Therefore, it is imperative to investigate the correlations between soil rhizobacterial communities and soil metabolites. Song et al. [20] revealed a significant association between starch and sucrose metabolism function and specific bacterial members within distinct metabolite pathways, providing further evidence for the influence of different bacterial members on regulating starch and sucrose metabolism function in a plant rhizosphere. In the present study, interaction network analysis found that bacterial taxa related to N cycling (*Nitrospira*) and Zn mobilization (*norank_f__norank_o__Vicinamibacterales* and *norank_f__Vicinamibacteraceae*), were significantly associated with carbohydrates, amino acids and the corresponding metabolic pathways, including amino acid metabolism and the metabolism of cofactors and vitamins, which were mainly involved in N metabolism and responses to Zn stress. This phenomenon supported the idea that soil microorganisms could influence the accumulation of soil metabolites and the relative metabolic process, subsequently altering soil ecological functions and even the growth of plants.

## 4. Materials and Methods

### 4.1. Experimental Design

The pot experiment was conducted at the Tai’an test base (E 117°5′4.391″, N 36°12′21″) of the Tea Research Institute, Shandong Academy of Agricultural Sciences. The experimental site exhibited a temperate monsoon climate, with an average annual precipitation of 697 mm and an annual mean temperature of 12.9 °C. The tested soil was collected from the tea plantation in Tai’an test base at a depth of 0–30 cm and was subsequently air-dried before being sieved through a 5 mm sieve to ensure homogeneity. Before the experiment, the basic properties of the collected soil were as follows—pH: 6.21, organic matter content: 18.2 g/kg, total nitrogen content: 1.09 g/kg, available phosphorus content: 23.3 mg/kg and available potassium content: 65 mg/kg.

The pot experiment was conducted on two-year-old plants of the tea cultivar “Zhongcha108” from September 2022 to September 2023, employing four fertilization treatments arranged in a completely randomized block design (Figure S8). Each treatment consisted of six biological replicates (6 pots), with a total of 24 pots. Five consistently growing tea plants were cultivated in each pot. Each pot had dimensions of a 32 cm upper diameter × 20 cm height × 18 cm bottom diameter and was uniformly filled with prepared soil weighing 7.5 kg. Urea was used for N fertilizer; zinc sulfate was used for Zn fertilizer. The treatments applied were as follows: (i) CK: control, involving the application of urea at a rate of 119 kg N/ha and ZnSO_4_·7H_2_O at a rate of 3 mg Zn/kg soil; (ii) N treatment: application of urea at a rate of 569 kg N/ha and ZnSO_4_·7H_2_O at a rate of 3 mg Zn/kg soil; (iii) Zn treatment: application of urea at a rate of 119 kg N/ha and ZnSO_4_·7H_2_O at a rate of 12 mg Zn/kg soil; and (iv) N+Zn treatment: application of urea at a rate of 569 kg N/ha and ZnSO_4_·7H_2_O at a rate of 12 mg Zn/kg soil. Urea was applied twice, in September (60%) and March (40%). Zinc sulfate was applied as a base fertilizer once in September 2022. Similarly, phosphorus was applied as superphosphate at a rate of 39.3 kg P/ha and potassium was applied as potassium sulfate at a rate of 99.6 kg K/ha in September 2022.

### 4.2. Soil-Sample Collection and Physicochemical-Properties Analysis

Rhizosphere soils were sampled based on the methods of Edwards et al. [43] with some minor modifications. Soil samples from the same treatment were evenly mixed, sieved and divided into four portions. One portion was air-dried, ground and passed through a 1 mm sieve for soil physicochemical properties (three replicates). The second portion was kept at 4 °C for the analysis of the microbial biomass element and enzyme activities (three replicates). The third portion was stored at −20 °C for DNA extraction (three replicates), while the remaining portion was frozen at −80 °C for metabolite extraction (six replicates). Soil properties and Zn concentrations were determined following the methods described by Bao [44]. Details of the analysis procedures can be found in the Supplementary Materials.

### 4.3. Measurements of MBC, MBN and Enzyme Activities in Rhizosphere Soil

The rhizosphere soil MBC and MBN contents were determined using chloroform fumigation followed by K_2_SO_4_ extraction with a TOC analyzer (multi-C/N 3100, Analytik Jena AG, Jena, Germany). Rhizosphere S-SC activity was determined according to Schinner and Von Mersi [45] based on a spectrophotometric measurement at 508 nm. Rhizosphere S-UE activity was determined according to Zhang et al. [46] using an ultraviolet spectrophotometer (Lambda 350 V-vis, PerkinElmer, Singapore). The measurement of rhizosphere S-ACP activity was conducted by using the disodium phenyl phosphate colorimetric method as Guan [47] depicted. Rhizosphere S-POD activity was determined with the autoxidation of the pyrogallol oxidation method. Rhizosphere S-PPO activity was measured based on the pyrogallol colorimetry method.

### 4.4. Soil DNA Extraction and 16S rRNA Gene Sequencing

Total genomic DNA of rhizosphere soil was extracted by the E.Z.N.A.^®^ soil DNA Kit (Omega Bio-tek, Norcross, GA, USA) following the manufacturer’s protocol. The V3-V4 region of the 16S rRNA gene for Illumina deep sequencing was amplified using primers 338F (5′-ACTCCTACGGGAGGCAGCA-3′) and 806R (5′-GGACTACHVGGGTWTCTAAT-3′) with barcodes as described by Liu et al. [48]. PCR amplification was performed on a T100 Thermal Cycler PCR thermocycler (BIO-RAD, Hercules, CA, USA). Purified amplicons were pooled in equimolar amounts and subjected to paired-end sequencing on an Illumina PE300/PE250 platform (Illumina, San Diego, CA, USA) following standard protocols provided by Majorbio Bio-Pharm Technology Co., Ltd. (Shanghai, China). All of the generated data were deposited in the National Center for Biotechnology Information (NCBI) Sequence Read Archive under Bioproject PRJNA1164154 with BioSample accession numbers SAMN43886391 to SAMN43886402. The Supplementary Materials provide detailed information regarding bacterial 16S amplification and pyrosequencing data processing.

### 4.5. Soil Metabolomics Analysis

The determination of rhizosphere soil metabolites was conducted through UHPLC-MS/MS analysis. The chromatographic separation of the metabolites was performed using a Thermo UHPLC system equipped with an ACQUITY BEH C18 column (100 mm × 2.1 mm i.d., 1.7 µm; Waters, Milford, CT, USA). The mass spectrometric data were collected using a Thermo UHPLC-Q Exactive Mass Spectrometer with an electrospray ionization (ESI) source operating in either positive or negative ion mode. Following UHPLC-MS/MS analyses, the raw data were imported into Progenesis QI 2.3 (Nonlinear Dynamics, Waters, USA) for peak detection and alignment. The compound identification of metabolites was established by matching MS/MS spectra with an in-house database containing available authentic standards. Further details can be found in the Supplementary Materials.

### 4.6. Statistical Analysis

Statistical analyses were conducted using SPSS 20.0 and Origin 8.5 based on two-way ANOVA analysis at a significance level of *p* < 0.05. Duncan’s Multiple Range Test (DMRT) was applied for multiple comparison procedures at 5% and 1% significance levels, respectively. Multivariate statistical analysis of soil bacterial community and soil metabolism was mainly conducted in the R (v4.1.0) package from Bioconductor on the Majorbio Cloud Platform (https://cloud.majorbio.com) (accessed on 10 November 2023). Network analysis within bacteria based on Pearson correlation matrices (|R| > 0.6, *p* < 0.05) was achieved on the top 100 OTUs of the total read count. The network topological properties of each sample were implemented in the subgraph function with the “igraph” package and were visualized in Gephi software (v0.9.2) [49]. The collinearity analysis of different marker metabolites and interactive networks between rhizobacterial taxa, metabolites and the metabolic pathway were analyzed using R.4.0.2 and were rendered by Cytoscape software (v3.10.1).

## 5. Conclusions

In this study, a pot experiment was employed to evaluate the effects of different N and Zn fertilization combinations on N availability, bacterial communities and metabolomics in the rhizosphere soil of tea plants. The findings demonstrated that N-Zn co-fertilization significantly affected soil total N contents and N availability by promoting MBN contents and the activities of S-UE. N-Zn co-fertilization improved the rhizosphere soil microenvironment of tea plants by directly and indirectly recruiting bacterial taxa associated with N cycling and Zn activation and by interfering with the soil lipid metabolism, amino acid metabolism and metabolism of cofactors and vitamins. And this modification might originate from the changes in soil pH and TN contents in response to N-Zn co-fertilization. Overall, all the above findings highlight the significant importance of optimizing combined applications of Zn and N fertilizers for the enhancement of N availability and the rhizosphere microenvironment of tea plants, which will provide a valuable understanding of the synergistic impact of nutrient elements for achieving nutrient use and management. Future studies should validate these findings under field conditions and explore long-term effects of N-Zn co-fertilization on soil health and tea quality. Additionally, the targeted manipulation of keystone microbial taxa or metabolites identified here may offer novel strategies for precision nutrient management in sustainable tea cultivation.

## Figures and Tables

**Table 1:** The physicochemical properties of rhizosphere soil of tea plants (*Camellia sinensis* L.) in response to different combinations of N and Zn fertilization.

	CK	N	Zn	N+Zn
pH (1:2.5)	6.17 ± 0.057 a	5.50 ± 0.018 c	5.94 ± 0.025 b	5.63 ± 0.054 c
Organic matter (g/kg)	19.5 ± 0.244 a	14.0 ± 0.033 c	16.7 ± 0.143 b	12.2 ± 0.303 d
CEC (cmol(+)/kg)	8.95 ± 0.748 a	8.06 ± 0.232 a	9.47 ± 0.729 a	8.81 ± 0.315 a
Total N (g/kg)	0.740 ± 0.025 b	1.17 ± 0.040 a	0.723 ± 0.067 b	1.13 ± 0.017 a
NO_3_^−^-N (mg/kg)	8.12 ± 0.094 b	19.5 ± 0.579 a	8.15 ± 0.101 b	20.3 ± 0.534 a
NH_4_^+^-N (mg/kg)	0.290 ± 0.012 b	0.970 ± 0.040 a	0.270 ± 0.017 b	0.953 ± 0.083 a
Available P (mg/kg)	22.0 ± 0.687 d	24.7 ± 0.444 c	30.0 ± 0.852 b	40.9 ± 0.632 a
Available K (mg/kg)	62.4 ± 0.590 b	70.4 ± 0.993 a	70.4 ± 0.995 a	69.3 ± 0.973 a
Total Zn (mg/kg)	124 ± 0.273 b	120 ± 0.705 b	142 ± 3.83 a	145 ± 3.76 a
DTPA-Zn (mg/kg)	2.19 ± 0.119 c	1.12 ± 0.039 d	8.04 ± 0.165 a	3.81 ± 0.096 b

Data represent the means ± standard errors (*n* = 3). Different letters in the same row indicate significant differences among all treatments at *p* < 0.05.

**Table 2:** The bacterial co-occurrence network topological properties in response to different combinations of N and Zn fertilization.

Topological Properties	CK	N	Zn	N+Zn
Number of Nodes	98	99	98	99
Number of Edges	235	347	230	315
Number of Positive Edges	111 (47.23%)	183 (52.74%)	137 (59.57%)	172 (54.60%)
Number of Negative Edges	124 (52.77%)	164(47.26%)	93 (40.43%)	143 (45.40%)
Average degree	4.796	7.01	4.694	6.364
Average clustering coefficient	0.736	0.783	0.745	0.75
Network diameter	15	13	13	17

## Data Availability

The datasets generated and analyzed during the current study are available from the corresponding author on reasonable request.

## Supplementary Materials

The following supporting information can be downloaded at: https://www.mdpi.com/article/10.3390/plants14121811/s1, Figure S1: Box plots of α-diversity indices including Sobs (a), Chao (b), Ace (c) Shannon (d), Simpson (e), Pielou_e (f) and Coverage (g) index of the soil rhizobacterial communities of tea plants in response to different combinations of N and Zn fertilization; Figure S2: Box plots of relative abundance of phyla *Proteobacteria* (a), *Acidobacteria* (b), *Firmicutes* (c), *Chloroflexi* (d) and *Patescibacteria* (e) in response to different combinations of N and Zn fertilization based on Kruskal-Wallis H test. ** indicates significance at *p* < 0.01. * indicates significance at *p* < 0.05; Figure S3: Differential abundance of bacterial orders in response to different combinations of N and Zn fertilization based on Kruskal-Wallis H test. Only bacterial orders whose relative abundance significantly (*p* < 0.05) changed are considered here; Figure S4: Differential abundance of bacterial genera in response to different combinations of N and Zn fertilization based on Kruskal-Wallis H test (a). Only bacterial genera whose relative abundance significantly (*p* < 0.05) changed are considered here. Box plots of relative abundance of genera *RB41* (b) and *Pseudolabrys* (c) in response to different combinations of N and Zn fertilization based on Kruskal–Wallis H test. ** indicates significance at *p* < 0.01. * indicates significance at *p* < 0.05; Figure S5: OPLS-DA score plots derived from metabolites between CK and N treatment (a). The expression volcano map of differential metabolites up and down regulation between CK and N treatment (b) (green dots represent down-regulated metabolites, red dots represent up-regulated metabolites and gray dots represent no-differential metabolites.). Differential abundance score of KEGG metabolic pathways between CK and N treatment (c). *** indicates significance at *p* < 0.001. ** indicates significance at *p* < 0.01. * indicates significance at *p* < 0.05; Figure S6: OPLS-DA score plots derived from metabolites between CK and Zn treatment (a). The expression volcano map of differential metabolites up and down regulation between CK and Zn treatment (b) (green dots represent down-regulated metabolites, red dots represent up-regulated metabolites and gray dots represent no-differential metabolites.). Differential abundance score of KEGG metabolic pathways between CK and Zn treatment (c). *** indicates significance at *p* < 0.001. ** indicates significance at *p* < 0.01. * indicates significance at *p* < 0.05; Figure S7: General profile of differential metabolites in rhizosphere soil of tea plants in response to different combinations of N and Zn fertilization; Figure S8: Photos of the pot experiment; Table S1: Pearson correlation analysis between chemical properties and enzyme activities in the rhizosphere soil of tea plants under different combinations of N and Zn fertilization; Table S2: Pearson correlation analysis between soil environmental variables and bacterial taxa (top 10 phyla) in the rhizosphere soil of tea plants under different combinations of N and Zn fertilization; Table S3: Pearson correlation analysis between soil environmental variables and bacterial taxa (top 10 orders) in the rhizosphere soil of tea plants under different combinations of N and Zn fertilization; Table S4: Pearson correlation analysis between soil environmental variables and bacterial taxa (top 10 genera) in the rhizosphere soil of tea plants under different combinations of N and Zn fertilization. References [50,51,52,53,54] are cited in the Supplementary Materials.
