# Supplementary material for: Integrated Microbiology and Metabolomics Analysis Reveal Responses of Soil Bacterial Communities and Metabolic Functions to N-Zn Co-Fertilization in the Rhizosphere of Tea Plants (Camellia sinensis L.)
Source: Plants (Basel). 2025 Jun 12;14(12):1811. doi: 10.3390/plants14121811 (PMC12197272; doi:10.3390/plants14121811)
Supplement: Supplementary file 1 [file plants-14-01811-s001.zip › plants-3628310-supplementary.pdf]

**Supplementary Materials**

**Supporting Methods**

**Analyses of rhizosphere soil physicochemical properties and elemental concentrations**

Soil pH values (soil:water = 1:2.5, m/m) were determined by calibrated pH meter (PB-10, Sartorius, Goettingen, Germany). Organic matter was measured according to the Walkley-Black wet digestion method. Soil CEC was determined using 1 mmol $L^{-1}$ $NH_4OAc$ extraction at pH 7.0 followed by titration. Total soil nitrogen was measured by Kjeldahl determination. Soil exch-$NH_4^+$-N and $NO_3^-$-N were extracted using 2 M KCl and then measured with a SAN++ Flow Injection Analyzer (SKALAR Ltd, Netherlands). Soil available phosphorus was determined by the ascorbic acid-ammonium molybdenum method. Soil available potassium was extracted with ammonium acetate and determined by flame photometry. For soil Zn concentration analysis, soil samples were digested with $HNO_3$/$HClO_4$/HF (5:1:1, v/v/v). And the available Zn was extracted with diethylene triamine pentaacetic acid (DTPA) (0.005 mmol/L DTPA, 0.01 mmol/L $CaCl_2$, and 0.1 mmol/L triethanolamine, pH = 7.3). Soil samples were extracted with agent above in a 1:2 soil/solution mixture, then filtered after shaking for 2 h. Zn concentration in the digested solutions and the extracts were determined using an Inductively Coupled Plasma Mass Spectrometer (ICP-MS, PlasmaQuant®MS, Germany). Sample replicates, reagent blanks, soil and plant standard reference material (GBW07429 and GBW100351, the National Research Center for Certified Reference Materials of China) were included in each batch of analysis to ensure the quality of analysis. The recovery of the standard for each element was 90-110%.

**Rhizosphere soil DNA extraction and 16S rRNA gene sequencing**

Rhizosphere soil bacterial DNA was extracted by the E.Z.N.A.® soil DNA Kit (Omega Bio-tek, Norcross, GA, U.S.) following the manufacturer's protocol and quantified with the NanoDrop2000

(Thermo Scientific, United States). To amplify the V3-V4 region of the 16S rRNA gene for Illumina deep sequencing, 338F (5'-ACTCCTACGGGAGGCAGCA-3') and 806R (5'-GGACTACHVGGGTWTCTAAT-3') primers with barcode were used by T100 Thermal Cycler PCR thermocycler (BIO-RAD, CA, USA). The PCR reaction mixture including 4 μL 5 × Fast Pfu buffer, 2 μL 2.5 mM dNTPs, 0.8 μL each primer (5 μM), 0.4 μL Fast Pfu polymerase, 10 ng of template DNA, and $ddH_2O$ to a final volume of 20 μL. After an initial denaturation at 95 °C for 3 min, amplification was performed by 27 cycles of incubations for 30 s at 95 °C, 30 s at 55 °C, and 45 s at 72 °C, followed by a final extension at 72 °C for 10 min. The PCR product was extracted from 2% agarose gel and purified using the PCR Clean-Up Kit (YuHua, Shanghai, China) according to manufacturer's instructions and quantified using Qubit 4.0 (Thermo Fisher Scientific, USA).

Purified amplicons were pooled in equimolar amounts and paired-end sequenced on an Illumina PE300/ PE250 platform (Illumina, San Diego, USA) according to the standard protocols by Majorbio Bio-Pharm Technology Co. Ltd. (Shanghai, China). All the generated data were submitted to the National Center for Biotechnology Information (NCBI) Sequence Read Archive under Bioproject PRJNA1164154 with BioSample accession numbers SAMN43886391 to SAMN43886402. Raw FASTQ files were de-multiplexed using an in-house perl script, and then quality-filtered by fastp version 0.19.6 [50] and merged by FLASH version 1.2.7 [51] with the following criteria: (i) the reads were truncated at any site receiving an average quality score of < 20 over a 50 bp sliding window, and the truncated reads shorter than 50 bp were discarded, reads containing ambiguous characters were also discarded; (ii) only overlapping sequences longer than 10 bp were assembled according to their overlapped sequence. The maximum mismatch ratio of overlap region is 0.2. Reads that could not be assembled were discarded; (iii) Samples were distinguished according to the barcode and primers, and the sequence direction was

adjusted, exact barcode matching, 2 nucleotide mismatches in primer matching. Then the optimized sequences were clustered into operational taxonomic units (OTUs) using UPARSE 7.1 [52-53] with 97% sequence similarity level. The most abundant sequence for each OTU was selected as a representative sequence. The OTU table was manually filtered, i.e., chloroplast sequences in all samples were removed. To minimize the effects of sequencing depth on alpha and beta diversity measure, the number of 16S rRNA gene sequences from each sample were rarefied to 20,000, which still yielded an average Good's coverage of 99.09%, respectively. The taxonomy of each OTU representative sequence was analyzed by RDP Classifier version 2.2 [54] against the 16S rRNA gene database (eg. Silva v138) using confidence threshold of 0.7. Bioinformatic analysis of the soil microbiota was carried out using the Majorbio Cloud platform (https://cloud.majorbio.com).

**Rhizosphere soil metabolomics analysis**

50 mg rhizosphere soil was accurately weighed, and the metabolites extracted using a 400 μL methanol:water (4:1, v/v) solution. The mixture was allowed to settle at −20°C and treated by High throughput tissue crusher Wonbio-96c (Shanghai wanbo biotechnology co., LTD) at 50 Hz for 6 min, then followed by vortex for 30 s and ultrasound at 40 kHz for 30 min at 5°C. The samples were placed at −20 ℃ for 30 min to precipitate proteins. After centrifugation at 13000 g at 4 ℃ for 15min, the supernatants were carefully transferred to sample vials for LC-MS/MS analysis.

Chromatographic separation of the metabolites was performed on a Thermo UHPLC system equipped with an ACQUITY BEH C18 column (100 mm × 2.1 mm i.d., 1.7 µm; Waters, Milford, USA). The samples were placed in a 4 °C automatic sampler during the entire analysis. Specifically, for each sample, a volume of 2.0 µL was loaded into the column and maintained at 40°C with a flow rate of 0.4 ml/min. The mobile phases consisted of 0.1% formic acid in water (solvent A) and 0.1% formic acid in

acetonitrile:isopropanol (1:1, v/v) (solvent B). The solvent gradient changed according to the following conditions: from 0 to 3 min, 95% (A): 5% (B) to 80% (A): 20% (B); from 3 to 9 min, 80% (A): 20% (B) to 5% (A): 95% (B); from 9 to 13 min, 5% (A): 95% (B) to 5% (A): 95% (B) ; from 13 to 13.1 min, 5% (A): 95% (B) to 95% (A): 5% (B), from 13.1 to 16 min, 95% (A): 5% (B) to 95% (A): 5% (B) for equilibrating the systems. The mass spectrometric data was collected using a Thermo UHPLC-Q Exactive Mass Spectrometer equipped with an electrospray ionization (ESI) source operating in either positive or negative ion mode. The optimal conditions were set as followed: Aus gas heater temperature, 400 °C; Sheath gas flow rate 40 psi; Aus gas flow rate 30 psi; ion-spray voltage floating (ISVF), −2800V in negative mode and 3500V in positive mode, respectively; Normalized collision energy, 20-40-60V rolling for MS/MS. Data acquisition was performed with the Data Dependent Acquisition (DDA) mode. The detection was carried out over a mass range of 70-1050 m/z.

After UPLC-TOF/MS analyses, the raw data were imported into the Progenesis QI 2.3 (Nonlinear Dynamics, Waters, USA) for peak detection and alignment. In the extracted ion features, only the variables with more than 80% of the nonzero measurement values in at least one group were kept. Compound identification of metabolites by MS/MS spectra with an in-house database was established by available authentic standards. Finally, A multivariate statistical analysis was performed using ropls (Version1.6.2, http://bioconductor.org/packages/release/bioc/html/ropls.html) R package from Bioconductor on Majorbio Cloud Platform (https://cloud.majorbio.com).

**References**

50. Chen, S.F.; Zhou, Y.Q.; Chen, Y.R.; Gu, J. Fastp: An ultra-fast all-in-one FASTQ preprocessor. *Bioinformatics* **2018**, *34*, 884-890.
51. Magoc, T.; Salzberg, S.L. FLASH: fast length adjustment of short reads to improve genome assemblies. *Bioinformatics* **2011**, *27*, 2957-2963.

52. Edgar, R.C. UPARSE: highly accurate OTU sequences from microbial amplicon reads. *Nat. Methods* **2013**, *10*, 996-998.
53. Stackebrandt, E.; Goebel, B.M. A place for DNA-DNA reassociation and 16S rRNA sequence analysis in the present species definition in bacteriology. *Int. J. Syst. Bacteriol.* **1994**, *44*, 846-849.
54. Wang, Q.; Garrity, G.M.; Tiedje, J.M.; Cole, J.R. Naive Bayesian classifier for rapid assignment of rRNA sequences into the new bacterial taxonomy. *Appl. Environ. Microbiol.* **2007**, *73*, 5261-5267.

## Supplementary Figures

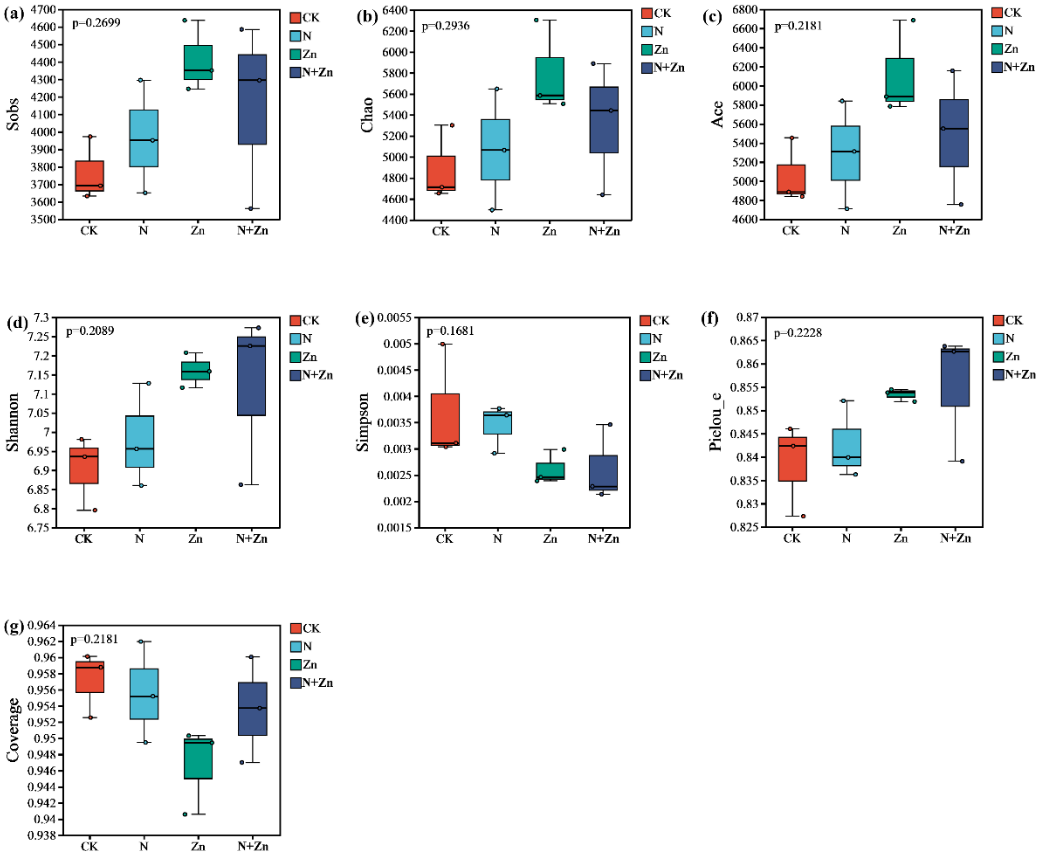


**Figure S1.** Box plots of α-diversity indices including Sobs (a), Chao (b), Ace (c) Shannon (d), Simpson (e), Pielou_e (f) and Coverage (g) index of the soil rhizobacterial communities of tea plants in response to different combinations of N and Zn fertilization.

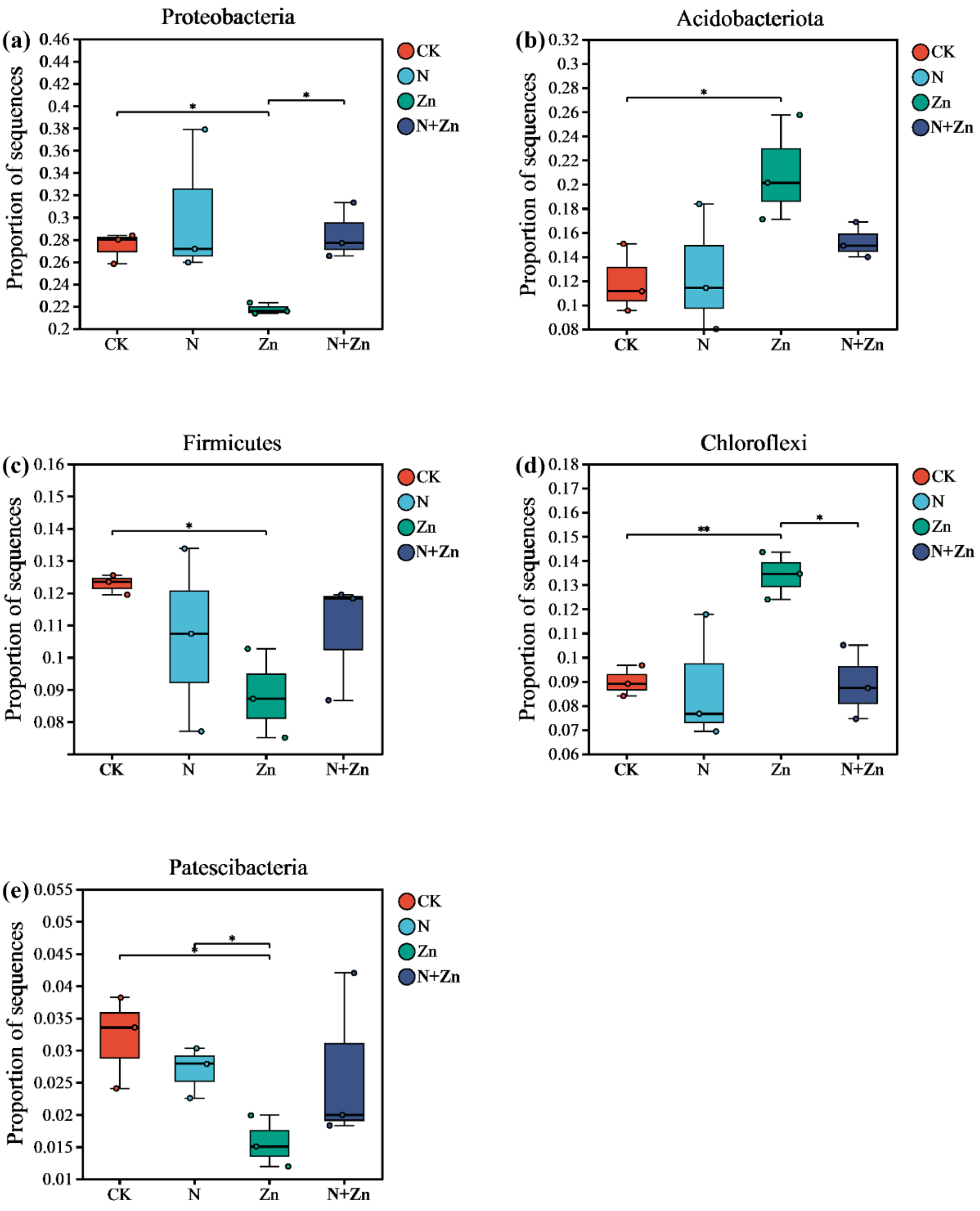


**Figure S2.** Box plots of relative abundance of phyla *Proteobacteria* (a), *Acidobacteria* (b), *Firmicutes* (c), *Chloroflexi* (d) and *Patescibacteria* (e) in response to different combinations of N and Zn fertilization based on Kruskal-Wallis H test. ** indicates significance at $p < 0.01$. * indicates significance at $p < 0.05$.

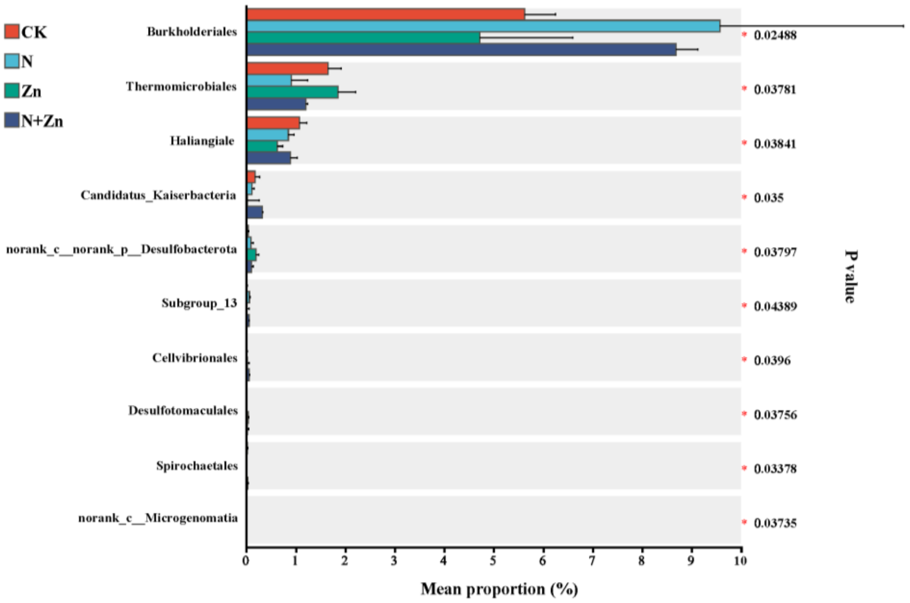


**Figure S3.** Differential abundance of bacterial orders in response to different combinations of N and Zn fertilization based on Kruskal-Wallis H test. Only bacterial orders whose relative abundance significantly ( $p < 0.05$) changed are considered here.

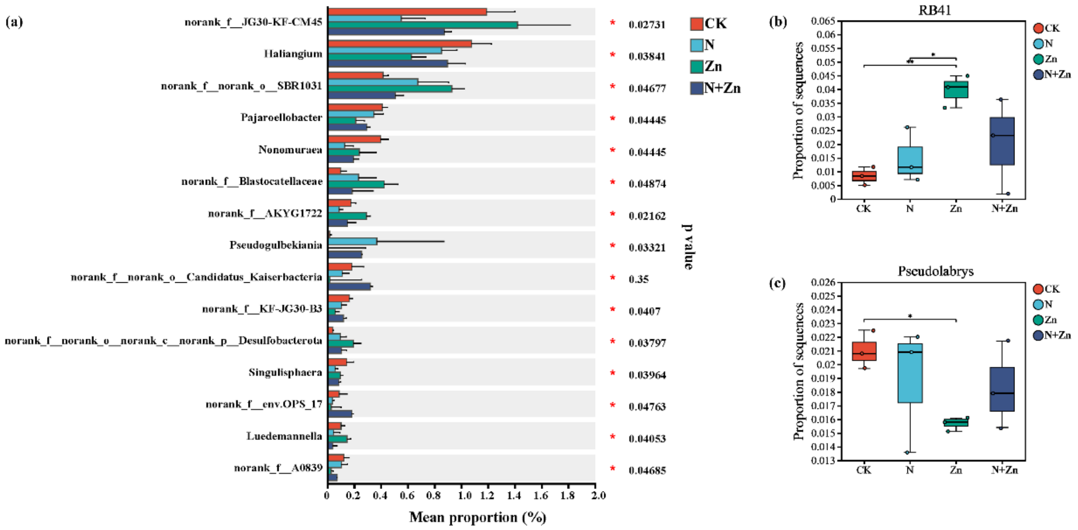


**Figure S4.** Differential abundance of bacterial genera in response to different combinations of N and Zn fertilization based on Kruskal-Wallis H test (a). Only bacterial genera whose relative abundance significantly ($p < 0.05$) changed are considered here. Box plots of relative abundance of genera *RB41* (b) and *Pseudolabrys* (c) in response to different combinations of N and Zn fertilization based on Kruskal-Wallis H test. ** indicates significance at $p < 0.01$. * indicates significance at $p < 0.05$.

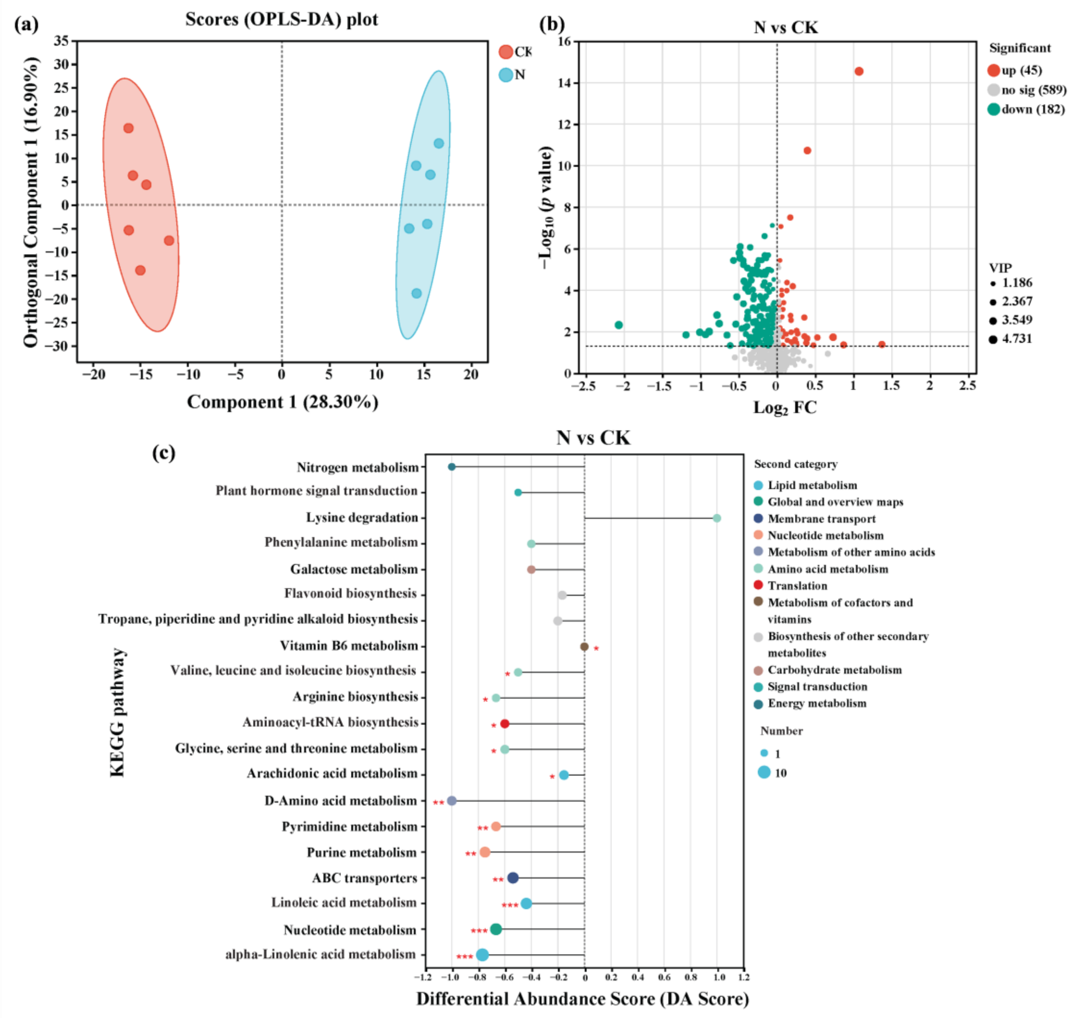


**Figure S5.** OPLS-DA score plots derived from metabolites between CK and N treatment (a). The expression volcano map of differential metabolites up and down regulation between CK and N treatment (b) (Green dots represent down-regulated metabolites, red dots represent up-regulated metabolites, and gray dots represent no-differential metabolites.). Differential abundance score of KEGG metabolic pathways between CK and N treatment (c). *** indicates significance at $p < 0.001$. ** indicates significance at $p < 0.01$. * indicates significance at $p < 0.05$.

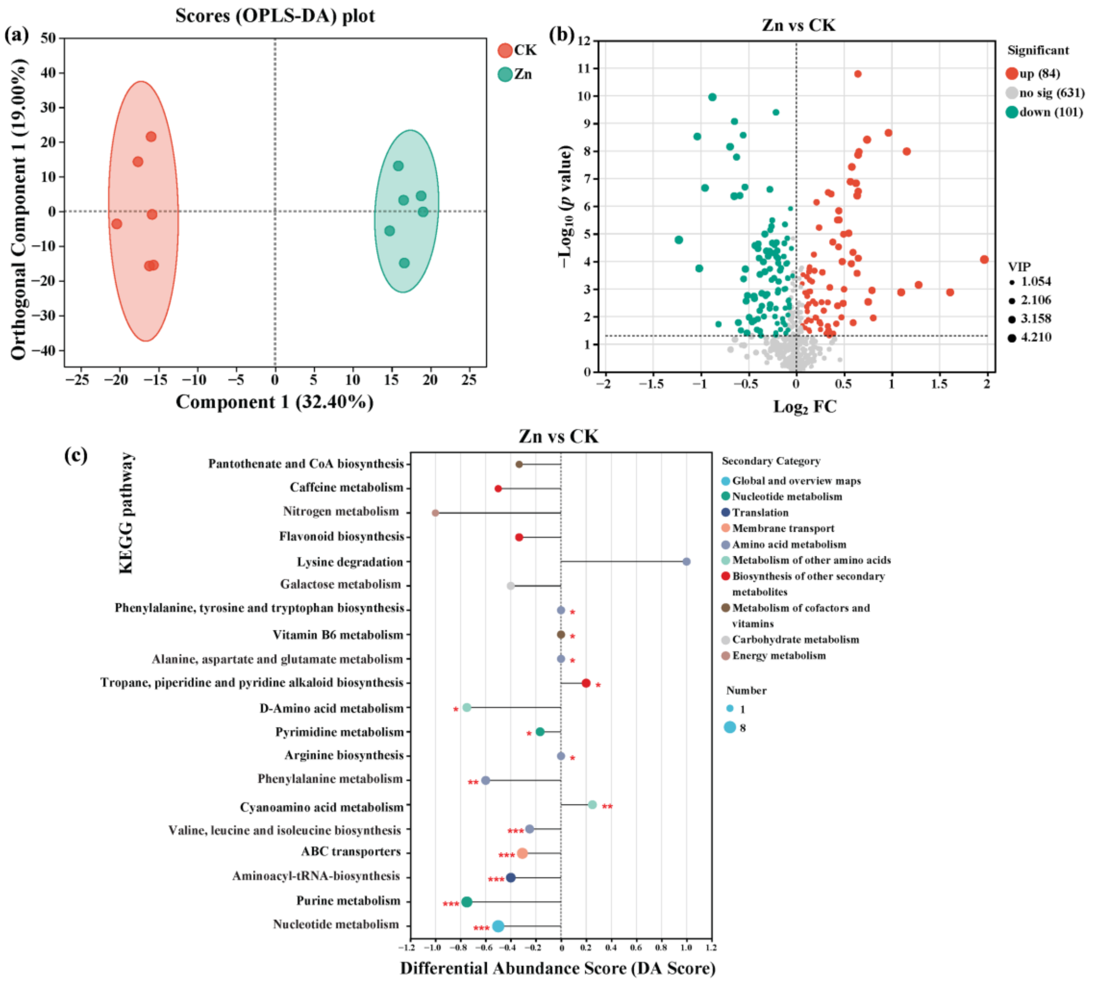


**Figure S6.** OPLS-DA score plots derived from metabolites between CK and Zn treatment (a). The expression volcano map of differential metabolites up and down regulation between CK and Zn treatment (b) (Green dots represent down-regulated metabolites, red dots represent up-regulated metabolites, and gray dots represent no-differential metabolites.). Differential abundance score of KEGG metabolic pathways between CK and Zn treatment (c). *** indicates significance at $p < 0.001$. ** indicates significance at $p < 0.01$. * indicates significance at $p < 0.05$.

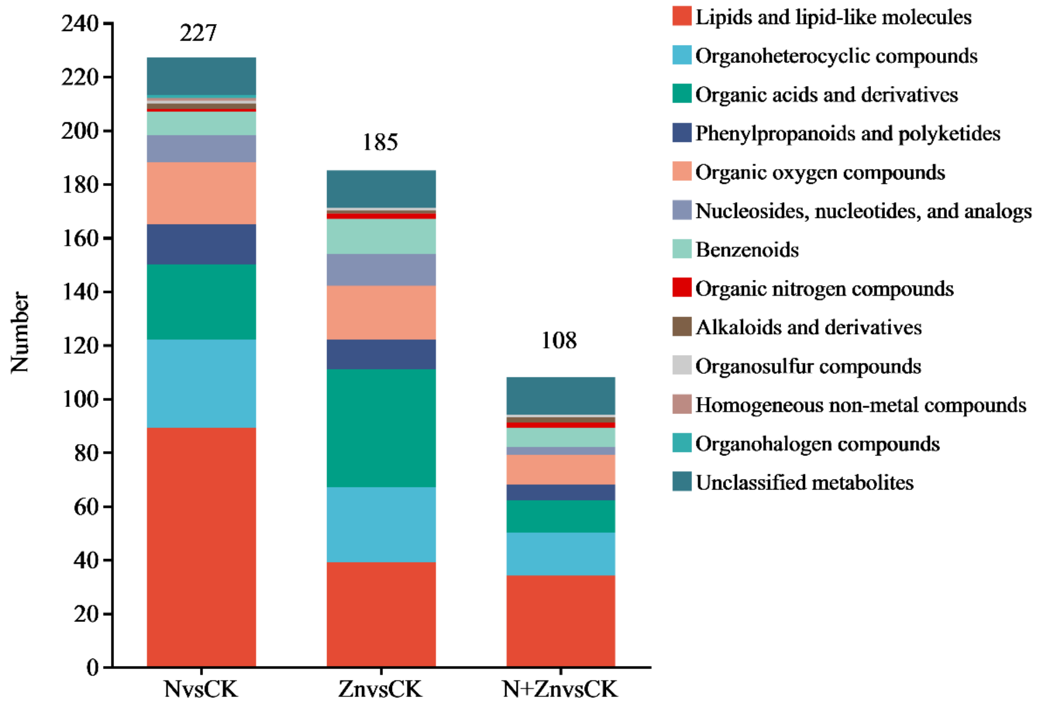


**Figure S7.** General profile of differential metabolites in rhizosphere soil of tea plants in response to different combinations of N and Zn fertilization.

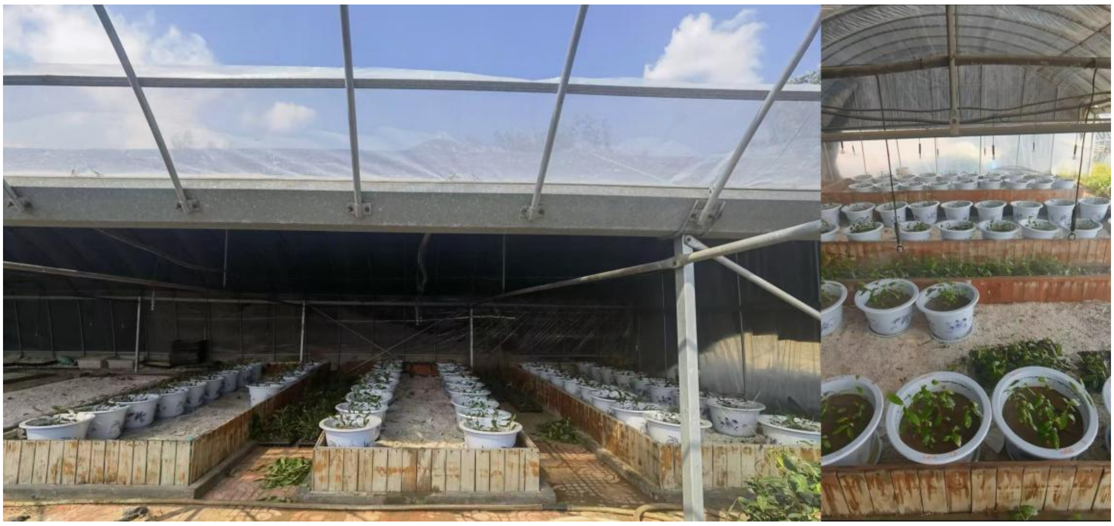

**Figure S8.** Photos of the pot experiment.

**Supplementary Tables**

**Table S1** Pearson correlation analysis between chemical properties and enzyme activities in the rhizosphere soil of tea plants under different combinations of N and Zn fertilization.

| | MBC | MBN | UE | ACP | SC | POD | PRO |
|---|---|---|---|---|---|---|---|
| pH | 0.835 | -0.825 | -0.564 | -0.890** | -0.773** | -0.895** | -0.211 |
| CEC | 0.354 | -0.270 | -0.626* | -0.320 | -0.485 | -0.284 | -0.527 |
| Organic matter | 0.969** | -0.954** | -0.322 | -0.909** | -0.591* | -0.973** | -0.054 |
| Total nitrogen | -0.837** | 0.860** | 0.725** | 0.953** | 0.822** | 0.854** | 0.433 |
| Available P | -0.736** | 0.773** | -0.038 | -0.797** | -0.381 | -0.911** | 0.166 |
| Available K | -0.614* | 0.546 | -0.007 | 0.515 | 0.192 | 0.659* | 0.117 |
| $NO_3^-$-N | -0.912** | 0.908** | 0.701* | 0.936** | 0.851** | 0.889** | 0.409 |
| $NH_4^+$-N | -0.868** | 0.861** | 0.659* | 0.944** | 0.831** | 0.917** | 0.327 |
| Total Zn | -0.305 | 0.344 | -0.730** | 0.110 | -0.385 | 0.303 | -0.657* |
| DTPA-Zn | 0.146 | -0.184 | -0.918** | -0.369 | -0.694* | -0.122 | -0.643* |

** indicates significance at $p < 0.01$. * indicates significance at $p < 0.05$.

**Table S2** Pearson correlation analysis between soil environmental variables and bacterial taxa (top 10 phyla) in the rhizosphere soil of tea plants under different combinations of N and Zn fertilization.

| | *Proteobacteria* | *Actinobacteriota* | *Acidobacteriota* | *Firmicutes* | *Chloroflexi* | *Gemmatimonadota* | *Myxococcota* | *Patescibacteria* | *Bacteroidota* | *Verrucomicrobiota* |
|---|---|---|---|---|---|---|---|---|---|---|
| pH | -0.356 | 0.499 | -0.019 | 0.122 | 0.294 | -0.267 | -0.261 | 0.125 | -0.537 | -0.416 |
| MBC | -0.319 | 0.538 | -0.003 | 0.118 | 0.265 | -0.487 | -0.451 | -0.004 | -0.494 | -0.364 |
| MBN | 0.371 | -0.479 | -0.084 | -0.056 | -0.312 | 0.509 | 0.495 | 0.038 | 0.397 | 0.287 |
| OC | -0.296 | 0.479 | -0.036 | 0.194 | 0.198 | -0.361 | -0.371 | 0.095 | -0.457 | -0.309 |
| TN | 0.657* | -0.333 | -0.336 | 0.003 | -0.555 | 0.293 | 0.274 | 0.204 | 0.465 | 0.286 |
| AP | 0.006 | -0.471 | 0.235 | -0.184 | 0.031 | 0.401 | 0.389 | -0.112 | 0.218 | 0.138 |
| AK | 0.050 | -0.200 | 0.184 | -0.498 | 0.316 | 0.042 | -0.132 | -0.212 | 0.280 | -0.003 |
| $NO_3^-$-N | 0.522 | -0.439 | -0.238 | 0.084 | -0.486 | 0.376 | 0.355 | 0.198 | 0.458 | 0.385 |
| $NH_4^+$-N | 0.557 | -0.415 | -0.261 | 0.112 | -0.496 | 0.292 | 0.245 | 0.260 | 0.421 | 0.365 |
| CEC | -0.596* | -0.084 | 0.685* | -0.220 | 0.415 | -0.017 | -0.122 | -0.555 | -0.349 | -0.031 |
| TZn | -0.442 | -0.235 | 0.469 | -0.321 | 0.459 | 0.244 | 0.298 | -0.440 | 0.018 | -0.002 |
| AZn | -0.693* | -0.081 | 0.740** | -0.524 | 0.771** | -0.112 | -0.178 | -0.616* | -0.134 | -0.166 |
| S-UE | 0.705* | -0.087 | -0.585* | 0.342 | -0.727** | 0.089 | 0.097 | 0.465 | 0.346 | 0.293 |
| S-ACP | 0.573 | -0.422 | -0.207 | -0.057 | -0.471 | 0.368 | 0.323 | 0.088 | 0.474 | 0.321 |
| S-SC | 0.561 | -0.558 | -0.287 | 0.405 | -0.700* | 0.276 | 0.310 | 0.297 | 0.629* | 0.640* |
| S-POD | 0.286 | -0.635* | 0.080 | -0.060 | -0.284 | 0.382 | 0.371 | -0.065 | 0.578* | 0.489 |
| S-PPO | 0.714** | 0.447 | -0.708** | -0.002 | -0.360 | -0.071 | -0.460 | 0.635* | -0.150 | -0.292 |

** indicates significance at $p < 0.01$. * indicates significance at $p < 0.05$.

**Table S3** Pearson correlation analysis between soil environmental variables and bacterial taxa (top 10 orders) in the rhizosphere soil of tea plants under different combinations of N and Zn fertilization.

| | ***Rhizobiales*** | ***Burkholderiales*** | ***Vicinamibacterales*** | ***Bacillales*** | ***Gaiellales*** | ***Micrococcales*** | ***Gemmatimonadales*** | ***norank_c__KD4-96*** | ***Micromonosporales*** | ***Sphingomonadales*** |
|---|---|---|---|---|---|---|---|---|---|---|
| pH | 0.337 | -0.669* | 0.230 | -0.258 | 0.248 | 0.264 | -0.251 | 0.098 | 0.742** | -0.531 |
| MBC | 0.476 | -0.558 | 0.189 | -0.242 | 0.158 | 0.601* | -0.496 | 0.132 | 0.715*** | -0.470 |
| MBN | -0.380 | 0.626* | -0.252 | 0.243 | -0.140 | -0.486 | 0.512 | -0.064 | -0.675* | 0.424 |
| OC | 0.399 | -0.576* | 0.156 | -0.212 | 0.146 | 0.453 | -0.372 | 0.030 | 0.753** | -0.423 |
| TN | -0.126 | 0.833*** | -0.529 | 0.325 | -0.022 | -0.207 | 0.326 | -0.150 | -0.501 | 0.347 |
| AP | -0.475 | 0.234 | 0.156 | 0.060 | -0.192 | -0.643* | 0.385 | 0.005 | -0.626* | 0.315 |
| AK | -0.281 | 0.372 | 0.068 | 0.027 | 0.024 | -0.301 | 0.052 | 0.394 | -0.618* | 0.391 |
| $NO_3^-$-N | -0.312 | 0.701* | -0.478 | 0.428 | -0.104 | -0.354 | 0.393 | -0.139 | -0.592* | 0.388 |
| $NH_4^+$-N | -0.274 | 0.716** | -0.520 | 0.482 | -0.090 | -0.304 | 0.308 | -0.126 | -0.567 | 0.377 |
| CEC | -0.092 | -0.530 | 0.755** | -0.374 | -0.290 | -0.019 | -0.113 | 0.018 | -0.076 | 0.086 |
| TZn | -0.388 | -0.249 | 0.499 | -0.206 | -0.145 | -0.444 | 0.213 | 0.194 | -0.432 | 0.137 |
| AZn | -0.262 | -0.509 | 0.834*** | -0.435 | -0.170 | -0.247 | -0.165 | 0.295 | -0.249 | 0.101 |
| S-UE | 0.115 | 0.648* | -0.760** | 0.441 | 0.068 | 0.127 | 0.131 | -0.290 | -0.019 | 0.110 |
| S-ACP | -0.201 | 0.792** | -0.411 | 0.275 | -0.092 | -0.315 | 0.375 | -0.172 | -0.619* | 0.476 |
| S-SC | -0.333 | 0.674* | -0.620* | 0.598* | -0.320 | -0.254 | 0.245 | -0.446 | -0.489 | 0.504 |
| S-POD | -0.541 | 0.584* | -0.191 | 0.344 | -0.303 | -0.545 | 0.360 | -0.139 | -0.828*** | 0.549 |
| S-PPO | 0.497 | 0.536 | -0.697* | 0.199 | 0.601* | 0.242 | 0.021 | 0.203 | 0.368 | -0.087 |

*** indicates significance at $p < 0.001$. ** indicates significance at $p < 0.01$. * indicates significance at $p < 0.05$.

**Table S4** Pearson correlation analysis between soil environmental variables and bacterial taxa (top 10 genera) in the rhizosphere soil of tea plants under different combinations of N and Zn fertilization.

| | *Bacillus* | *norank_f__norank_o__Vicinamibacterales* | *norank_f__norank_o__Gaiellales* | *norank_f__norank_o__norank_c__KD4-96* | *RB41* | *Pseudarthrobacter* | *norank_f__Vicinamibacteraceae* | *Pseudolabrys* | *Sphingomonas* | *Nitrospira* |
|---|---|---|---|---|---|---|---|---|---|---|
| pH | -0.339 | 0.334 | 0.193 | 0.098 | -0.091 | 0.184 | 0.110 | 0.190 | -0.429 | -0.337 |
| MBC | -0.326 | 0.289 | 0.106 | 0.132 | -0.063 | 0.541 | 0.064 | 0.200 | -0.369 | -0.439 |
| MBN | 0.306 | -0.327 | -0.086 | -0.064 | -0.005 | -0.411 | -0.154 | -0.072 | 0.315 | 0.481 |
| OC | -0.295 | 0.244 | 0.113 | 0.030 | -0.093 | 0.382 | 0.051 | 0.207 | -0.316 | -0.370 |
| TN | 0.367 | -0.589* | 0.088 | -0.150 | -0.293 | -0.161 | -0.440 | 0.132 | 0.179 | 0.405 |
| AP | 0.113 | 0.113 | -0.198 | 0.005 | 0.246 | -0.578* | 0.204 | -0.243 | 0.282 | 0.388 |
| AK | 0.170 | -0.030 | 0.021 | 0.394 | 0.309 | -0.279 | 0.162 | -0.287 | 0.345 | -0.087 |
| $NO_3^-$-N | 0.482 | -0.544 | -0.003 | -0.139 | -0.224 | -0.293 | -0.381 | 0.020 | 0.267 | 0.424 |
| $NH_4^+$-N | 0.532 | -0.574 | 0.027 | -0.126 | -0.293 | -0.247 | -0.434 | 0.078 | 0.262 | 0.373 |
| CEC | -0.389 | 0.797** | -0.428 | 0.018 | 0.533 | 0.040 | 0.672* | -0.222 | 0.217 | -0.263 |
| TZn | -0.144 | 0.474 | -0.272 | 0.194 | 0.552 | -0.397 | 0.508 | -0.457 | 0.179 | 0.059 |
| AZn | -0.371 | 0.810** | -0.318 | 0.295 | 0.767** | -0.230 | 0.820** | -0.526 | 0.218 | -0.267 |
| S-UE | 0.425 | -0.780** | 0.219 | -0.290 | -0.597* | 0.124 | -0.702* | 0.341 | -0.022 | 0.266 |
| S-ACP | 0.334 | -0.475 | -0.020 | -0.172 | -0.156 | -0.255 | -0.321 | 0.042 | 0.316 | 0.377 |
| S-SC | 0.624* | -0.689* | -0.180 | -0.446 | -0.283 | -0.197 | -0.509 | -0.014 | 0.413 | 0.504 |
| S-POD | 0.431 | -0.282 | -0.247 | -0.139 | 0.120 | -0.461 | -0.078 | -0.287 | 0.465 | 0.454 |
| S-PPO | 0.218 | -0.654* | 0.731** | 0.203 | -0.766** | 0.156 | -0.722** | 0.711** | -0.223 | -0.343 |

** indicates significance at $p < 0.01$. * indicates significance at $p < 0.05$.
